# Atemwegsmanagement bei penetrierendem zervikofazialem Trauma: Bedeutung der fiberoptischen Intubation bei erwartet schwierigem Atemweg

**DOI:** 10.1007/s00101-026-01749-0

**Published:** 2026-10-12

**Authors:** Ladina Jungck, Christoph Konrad, Thomas Arnold, Michèle Schöb, Robert Herr

**Affiliations:** 1 Klinik für Anästhesie, Luzerner Kantonsspital, Kantonsspital 37, 6004 Luzern, Schweiz; 2 Universität Luzern, Luzern, Schweiz

**Keywords:** Traumatische Atemwegsverletzung, Endotracheale Intubation, Flexible Endoskopie, Halsverletzung, Notfallintubation, Traumatic airway injury, Endotracheal intubation, Flexible endoscopy, Neck injury, Emergency intubation

## Abstract

Penetrierende Verletzungen in Gesichts- und Halsbereich stellen eine besondere Herausforderung für das Atemwegsmanagement dar. Bei einem 54-jährigen Patienten mit penetrierender Unterkieferverletzung durch einen Industrieroboterarm wurde aufgrund eines erwartet schwierigen Atemwegs eine wache nasale fiberoptische Intubation (FOI) in sitzender Position durchgeführt. Der Fall unterstreicht die Bedeutung dieses Verfahrens in Situationen mit erwartet schwierigem Atemweg.

## Abstract (en)

Penetrating injuries of the face and neck region pose a particular challenge for airway management. In a 54-year-old male patient with a penetrating mandibular injury caused by an industrial robotic arm, an awake nasal fiberoptic intubation (FOI) was performed in the sitting position because a difficult airway was anticipated. This case highlights the importance of this procedure in situations where a difficult airway is expected.

## Fallvorstellung

Ein 54-jähriger Patient ohne relevante Vorerkrankungen erlitt einen schweren Arbeitsunfall durch einen Roboterarm. Der Arm des Roboters bohrte sich mit vorangehender Zange von rechts lateral in den Kopf und Hals des Patienten. Der Patient war zunächst eingeklemmt und wurde durch Mitarbeitende befreit. Der Hauptarm des Roboters konnte durch Werkstattmitarbeitende entfernt werden. Es verblieb ein etwa 2 kg schweres Metallteil mit vorangehender Zange, das ca. 8 cm tief in den Unterkieferstrukturen verankert war und ca. 20 cm aus dem Körper herausragte. Der Patient hielt den Fremdkörper eigenständig fest und liess keine Unterstützung zu.

Beim Eintreffen des Rettungsdienstes präsentierte sich ein potenziell bedrohter Atemweg. Mund und Nase waren blutig; die Mundöffnung war nur etwa 1 cm möglich. Unter nasaler Sauerstoffapplikation (4 l/min) zeigte der Patient eine suffiziente Spontanatmung. Der Patient war kaltschweissig und tachykard. Neurologisch zeigte sich eine psychisch sehr angespannte, unkooperative und verbal aggressive Verfassung. Aufgrund des zu diesem Zeitpunkt nicht vorhersehbaren Verletzungsmusters, der vorhandenen ausreichenden Spontanatmung, der begrenzten personellen und materiellen präklinischen Ressourcen sowie der geringen Distanz zum Traumazentrum wurde auf eine präklinische Atemwegssicherung verzichtet. Die i.v.-Analgesie und Anxiolyse erfolgten fraktioniert mit einer Kumulativdosis von 0,3 mg Fentanyl, 20 mg Esketamin und 2 mg Midazolam. Der Transport erfolgte in den Schockraum des nächsten Traumazentrums.

**Abb. 1:**
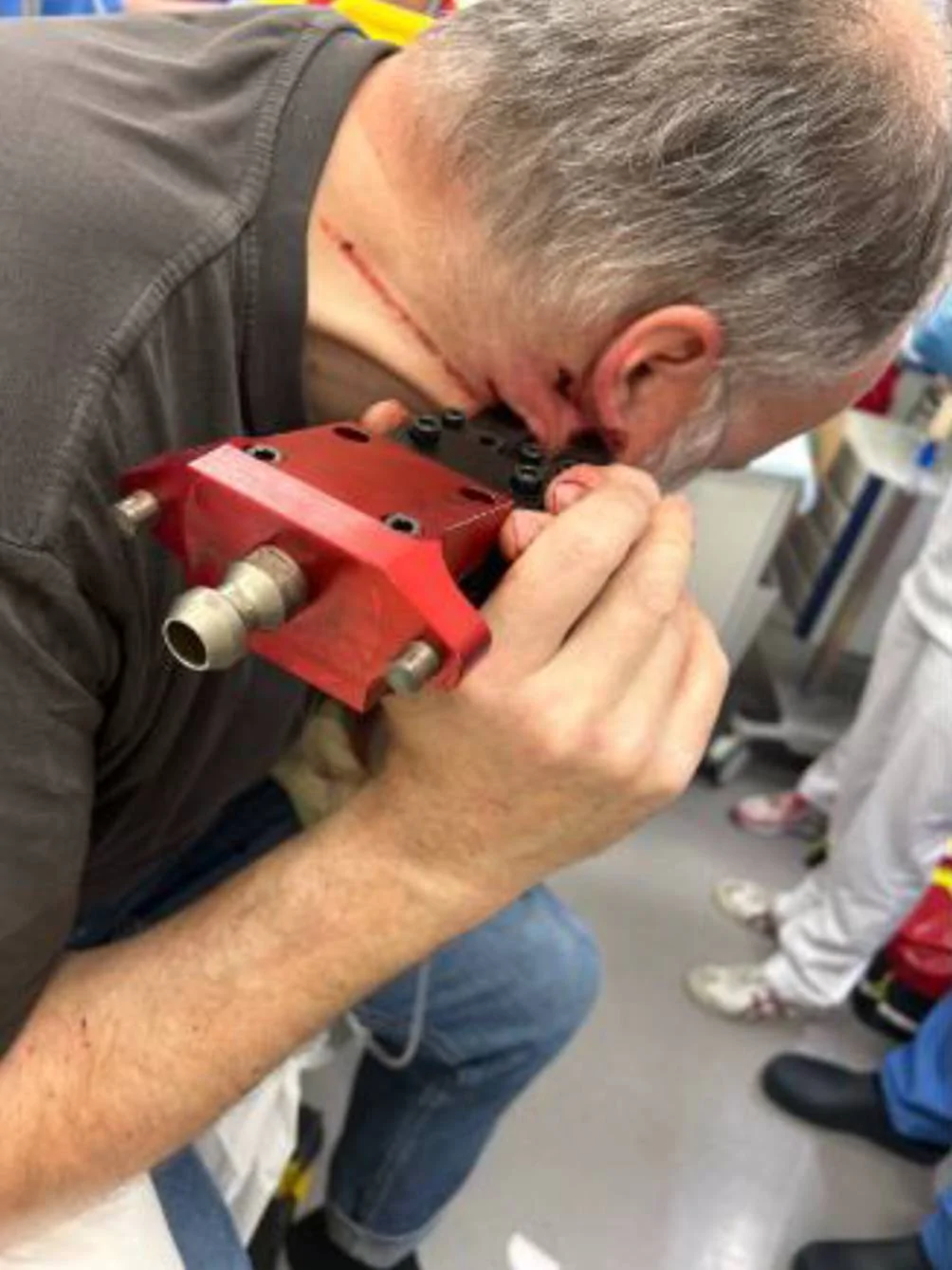
Eindringender Roboterarm im Bereich des rechten Unterkieferwinkels und der seitlichen Halsregion. Der Patient sitzt nach vorne geneigt und hält den Fremdkörper zur Stabilisierung fest. Eine schriftliche Einwilligung des Patienten zur Publikation liegt vor

Beim Eintreffen im Schockraum präsentierte sich der Patient sitzend auf der Trage des Rettungsdienstes, mit der Metallzange eines Roboterarms im rechten Hals‑/Kieferwinkel, den Fremdkörper mit beiden Händen fixierend (Abb. 1). Die strukturierte Erstbeurteilung erfolgte nach dem xABCDE-Schema. Es zeigten sich keine kritischen externen Blutungen. Die Atemwege waren bei erhaltener Spontanatmung, soweit beurteilbar, frei, wobei aufgrund des Unfallmechanismus eine Instabilität der Halswirbelsäule nicht ausgeschlossen werden konnte. Die Auskultation der Lunge ergab eine seitengleiche Belüftung bei einer peripheren Sauerstoffsättigung von 98 % unter Sauerstoffinsufflation (4 l/min, nasal). Hämodynamisch präsentierte sich der Patient hypertensiv bei normokarder Herzfrequenz. Neurologisch war der Patient mit einem Wert der Glasgow Coma Scale von 15 Punkten wach und kontaktierbar, jedoch ausgeprägt unkooperativ. Die Pupillen waren prompt und isokor lichtreagibel, die Extremitäten soweit beurteilbar frei beweglich. Hinweise auf ein fokal-neurologisches Defizit waren nicht vorhanden. Die Ganzkörperinspektion ergab keine Hinweise auf weitere Traumafolgen. Im Schockraum waren alle relevanten Fachdisziplinen anwesend: Notfallmedizin, Anästhesie, Hals-Nasen-Ohrenheilkunde, Mund-Kiefer-Gesichtschirurgie, Neurochirurgie und Gefässchirurgie.

Angesichts der anatomischen Verzerrung durch das penetrierende Trauma, der stark limitierten Mundöffnung, der unmöglichen Vorhersage der Atemwegsanatomie und des Risikos einer Atemwegsobstruktion bei Narkoseeinleitung wurde die Entscheidung getroffen, die primäre Atemwegssicherung nasal fiberoptisch wach bei sitzendem Patienten durchzuführen. Für die wache fiberoptische Intubation wurde folgendes Equipment vorbereitet: flexibles Bronchoskop mit 5,0 mm Aussendurchmesser, Spiralfedertubus mit Innendurchmesser 6,5 mm sowie Spiralfedertuben in kleineren Grössen als Back-up, topische Anästhesie sowie ein Absaugungssystem. Eine Überwachung erfolgte mittels der Pulsoxymetrie über einen Fingerclip sowie EKG; die Blutdruckmanschette wurde weiterhin nicht toleriert. Für die Intubation stand das HNO-Team mit ausgedecktem Koniotomie- sowie Tracheotomieset (Melker-Koniotomie-Set, Instrumentarium zur chirurgischen Tracheotomie) während der gesamten fiberoptischen Intubation im Schockraum bereit, um im Bedarfsfall eine unmittelbare chirurgische Atemwegssicherung zu gewährleisten.

## Wache fiberoptische Intubation

Der Patient befand sich sitzend auf der Trage des Rettungsdienstes und tolerierte aufgrund von Schmerz und Angst keine Lageänderung. Er hielt den Fremdkörper verkrampft fest und zeigte sich sehr ängstlich und schwer zu motivieren, er traute niemandem zu, den Roboterarm halten zu können. Die topische Vorbereitung des nasalen Atemwegs erfolgte gemäss lokaler Standard Operating Procedure (SOP) [1] mit einer Mischung von Lidocain, 4 %ig, und Cocain, 10 %ig, über ein Mucosal Atomization Device (2,5 ml Lidocain, 4 %ig, entsprechend 100 mg Lidocain, 0,5 ml Cocain, 10 %ig, entsprechend 50 mg Cocain). Während der Fiberendoskopie erfolgte eine weitere topische Anästhesie mittels „Spray-as-you-go“-Technik, hierfür wurde Lidocain, 4 %ig, über die Optik verabreicht (2 ml Lidocain, 4 %ig, entsprechend 80 mg Lidocain). Im Verlauf der topischen Anästhesie und nach Etablierung einer titrierten Remifentanildauerinfusion (TCI-Modell nach Minto [2], Ziel-Effekt-Konzentration (Ce) 0,5–1,5 ng/ml), bei der eine leichte Sedierung mit Erhalt von Kontaktierbarkeit, Spontanatmung und Schutzreflexen angestrebt wurde, besserte sich die Kooperation zunehmend. In Kombination mit kontinuierlichem verbalem Zureden und Motivieren sowie positiver Suggestion konnte so eine für die Durchführung der Wachintubation ausreichende Kooperation erreicht werden. Aufgrund der stark limitierten Mundöffnung von weniger als 1 cm wurde trotz noch unklarem Verletzungsmuster die Entscheidung zur nasalen Intubation getroffen. Die sitzende Position des Patienten wurde beibehalten; eine Lageänderung wurde nicht toleriert. Während der Fiberendoskopie des nasalen Atemwegs kam es zum Erbrechen, es wurde über einen Absaugkatheter enoral sowie transnasal abgesaugt. Die anschliessende Neupositionierung der Fiberoptik gelang problemlos. Klinisch zeigten sich erhaltene Schutzreflexe, es kam während des Erbrechens mehrmals zu Husten. Eine Aspiration konnte später fiberoptisch ausgeschlossen werden. Die erfolgreiche Sondierung bis in die Trachea wurde unter direkter Visualisierung durchgeführt, gefolgt vom Vorschub des Tubus nach endotracheal. Die Fiberendoskopie zeigte eine freie nasale Passage links, kein Hinweis auf endonasale Verletzungen, der Epipharynx war frei, die Rachenhinterwand zeigte eine Blutspur, aber keine offenen Verletzungen. Der Larynx präsentierte sich mit reizlosen Schleimhäuten, keine Rötung oder Schwellung, die Epiglottis war schlank, die Stimmlippen und Trachea waren unauffällig. Die korrekte Tubuslage wurde kapnographisch bestätigt, anschliessend erfolgten die i.v.-Narkoseinduktion sowie Ergänzung des Monitorings, inklusive erstmalige Blutdruckmessung. Die Atemwegssicherung wurde durch zwei erfahrene Fachärzte der Anästhesie durchgeführt.

## Diagnostik, Therapie und Verlauf

**Abb. 2:**
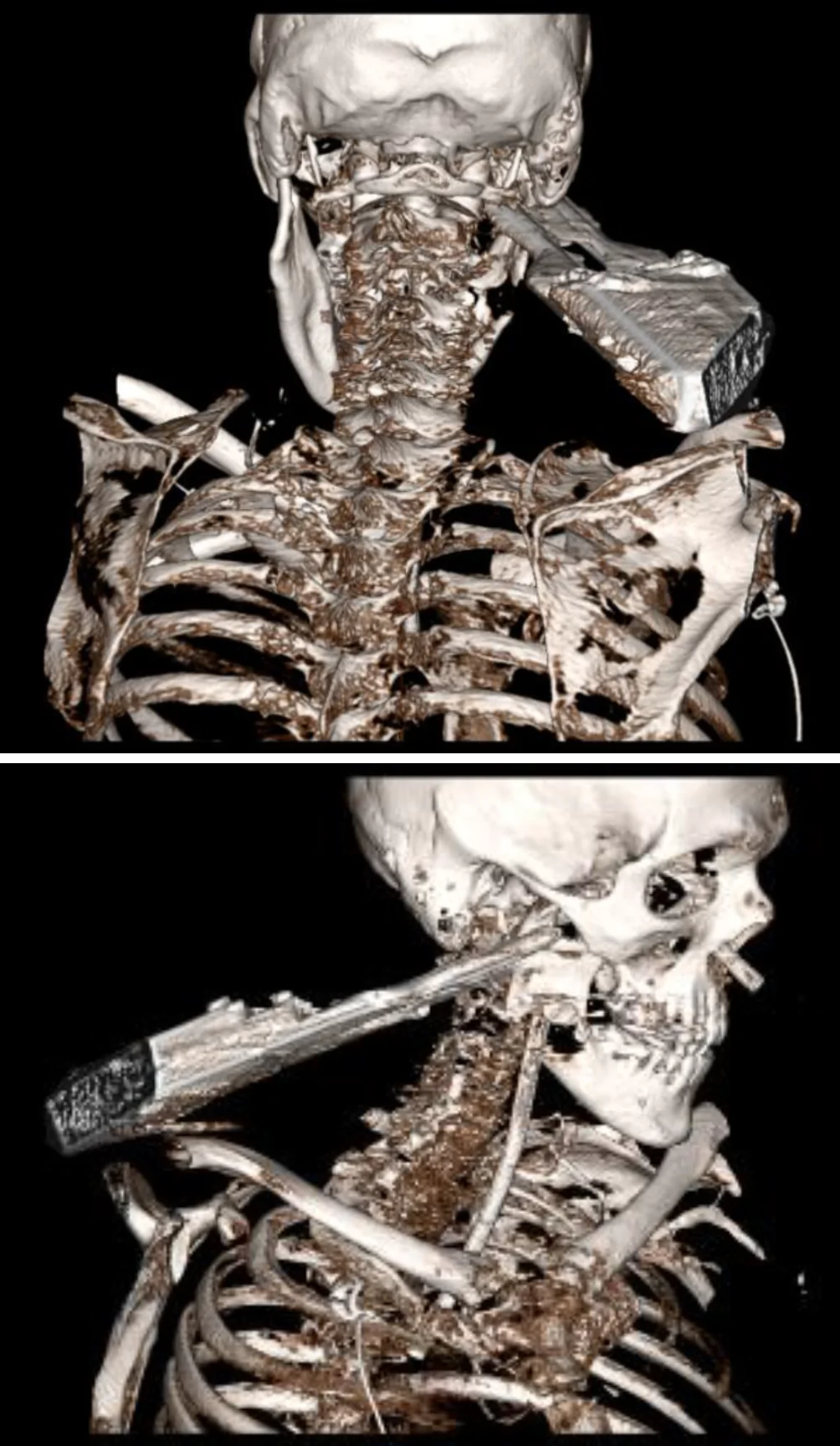
CT-Untersuchung nach wacher FOI mit Darstellung des penetrierenden Roboterarms

In der im Anschluss durchgeführten Computertomographie von Schädel und Hals zeigte sich eine bukkale Fremdkörperperforation rechts mit den Spitzen des Fremdkörpers am Os sphenoidale und lateral des Ramus mandibulae (Abb. 2). Es fanden sich eine Fraktur des Collum mandibulae rechts mit medialer Luxation sowie eine gering dislozierte Fraktur der Lamina lateralis des Processus pterygoideus rechts. Zusätzlich wurde eine Nasenbeinfraktur rechts diagnostiziert. Hinsichtlich Verletzungen vaskulärer Strukturen ergab sich kein eindeutiger Hinweis. Es zeigten sich keine intrakranielle Blutung und keine Kalottenfraktur, die Halswirbelsäule stellte sich intakt dar. Hinweise auf eine Trachealverletzung waren nicht vorhanden.

Es erfolgte die Verlegung in den Operationssaal zur kontrollierten Extraktion des Fremdkörpers. Postoperativ wurde der Patient auf die Intensivstation verlegt. Die Extubation konnte problemlos durchgeführt und der Patient am ersten postoperativen Tag auf die Normalstation verlegt werden. Der weitere Verlauf gestaltete sich komplikationslos. Am fünften postoperativen Tag konnte der Patient entlassen werden, es bestanden weiterhin eine Fazialisparese rechts sowie eine Hypästhesie des zweiten und dritten Astes des N. trigeminus rechts. Die Osteosynthese des Unterkiefers erfolgte im Verlauf.

## Diskussion

Bereits die präklinische Entscheidung gegen eine Atemwegssicherung stellt einen zentralen Learning Point dieses Falles dar. Bei erhaltener suffizienter Spontanatmung, unvorhersehbarem Verletzungsmuster und limitierten Ressourcen vor Ort wurde das Risiko einer präklinischen Atemwegsintervention höher eingeschätzt als deren Nutzen. Dies steht im Einklang mit der Literatur, die bei der prähospitalen Atemwegssicherung bei Traumapatienten eine individualisierte Entscheidungsfindung unter Berücksichtigung von Patienten‑, Team- und Umgebungsfaktoren mit Fokus auf adäquate Oxygenierung und Ventilation befürwortet [3]. Der präklinische Verzicht auf eine Atemwegssicherung ermöglichte den unmittelbaren Transport ins Traumazentrum, wo das Team im Schockraum vor der Herausforderung stand, für dieses komplexe Verletzungsmuster mit multiplen Prädiktoren für einen schwierigen Atemweg eine strukturierte, sichere Atemwegsstrategie festzulegen.

Die Wahl der wachen nasalen FOI in diesem Fall basierte auf mehreren etablierten Indikationen: Der Patient präsentierte sich mit einer Mundöffnung von weniger als 1 cm, was sowohl die direkte Laryngoskopie als auch die Videolaryngoskopie praktisch nicht durchführbar machte. Zusätzlich bestand durch den penetrierenden Fremdkörper in unmittelbarer Nähe zu den grossen Halsgefässen ein Risiko der Atemwegsverlegung durch Blutung oder ein Hämatom. In unserem Fall war die Erhaltung der Spontanatmung und der Schutzreflexe essenziell, da das Ausmaß der Atemwegsverletzung unbekannt war und es sich um einen nichtnüchternen Traumapatienten handelte. Das unklare Verletzungsmuster der Schädelbasis und des Mittelgesichts im Hinblick auf den nasalen Zugangsweg wurde als vertretbares Risiko bewertet, es wurde bewusst die fiberoptisch kontrollierte, nichtblinde Vorschubtechnik unter fortlaufender visueller Kontrolle gewählt. Zudem tolerierte der Patient keine Lageänderung, sodass weder eine Computertomographie zur vorhergehenden Abklärung des Verletzungsmusters noch eine primäre Tracheotomie im Liegen möglich waren; die wache nasale FOI am sitzenden Patienten stellte somit den einzig gangbaren Weg zur Atemwegssicherung dar.

Die wache nasale FOI weist eine sehr hohe Erfolgsrate von in der Literatur beschriebenen 98–99 % bei gleichzeitig niedriger Komplikationsrate auf [4–7]. Zu beachten ist ein mitunter erhöhter Zeitaufwand [8]. In einer Situation, wie sie bei unserem Patienten vorlag, wurde dieser Zeitaufwand akzeptiert und durch die erhöhte Sicherheit gerechtfertigt. Eine Versagensrate von 3,5 %, am häufigsten aufgrund von Tubusdislokation nach Narkoseeinleitung, Patientenunruhe, Unmöglichkeit, den Tubus vorzuschieben, und Atemwegsblutung, ist beschrieben [9]. Gerade bei fazialem Trauma mit vorbestehender Blutung im Atemweg kann die Visualisierung erheblich erschwert sein. Zusätzlich kann die Bronchoskopie selbst Blutungen induzieren, insbesondere bei nasaler fiberoptischer Intubation wurden Blutungen in bis zu 10 % beobachtet [10].

Ein weiterer zentraler Aspekt der Nutzen-Risiko-Abwägung war das Aspirationsrisiko bei nichtnüchternem Traumapatienten. Im Gegensatz zur Rapid Sequence Induction, bei der die Schutzreflexe bei der Narkoseeinleitung aufgehoben werden, bleiben bei der wachen FOI Bewusstsein, Spontanatmung und Schutzreflexe erhalten, sodass bei einer Regurgitation eine Aspiration durch aktives Husten verhindert wird und zusätzlich mittels Absaugung unterstützt werden kann [11]. Dies bestätigte sich in vorliegenden Fall: Trotz des während der Fiberendoskopie aufgetretenen Erbrechens verhinderten die erhaltenen Schutzreflexe in Kombination mit sofortiger oraler und transnasaler Absaugung eine Aspiration, was im Anschluss fiberoptisch bestätigt werden konnte.

Die Beherrschung der wachen nasalen FOI erfordert strukturiertes Training und eine ausreichende Zahl an Prozeduren, um Kompetenz zu erreichen [12], gleichzeitig wird eine besorgniserregende Unterexposition dokumentiert. Eine nationale Umfrage unter Schweizer Anästhesist:innen von 2023 (Mackert et al.) zeigt, dass die Mehrheit der praktizierenden Anästhesist:innen die Expertise zur Durchführung einer fiberoptischen Wachintubation für ihre tägliche Praxis als wichtig erachtet, jedoch fast ein Drittel die eigene Fähigkeit in dieser Technik als unzureichend bewertet, wodurch eine grosse Sicherheitslücke entsteht [13]. Die Technik der fiberoptischen Intubation für das sichere Management des erwartet schwierigen Atemweges wird daher zu Recht als unverzichtbar angesehen [14]. Unser Fall unterstreicht die Notwendigkeit einer ausreichenden Exposition und strukturierten Ausbildung in der fiberoptischen Intubation im klinischen Alltag, obwohl die Videolaryngoskopie meistens im Vordergrund steht.

Ein zentraler Aspekt des Atemwegsmanagements bei penetrierendem Gesichts- und Halstrauma ist die Antizipation eines möglichen Versagens der primären Intubationsstrategie [15]. Im vorliegenden Fall wurde die wache nasale FOI mit dem Vorteil gewählt, dass die eigene Atemwegsdurchgängigkeit bei erhaltenem Bewusstsein und Spontanatmung aufrechterhalten wird und Zeit für alternative Strategien gewonnen werden können. Der Wechsel auf eine orale fiberoptische Intubation oder Videolaryngoskopie war in unserem Fall bei extrem kleiner Mundöffnung <1 cm keine Option, ebenso wenig der Einsatz einer supraglottischen Atemwegshilfe. In der Situation eines „Cannot Intubate, Cannot Oxygenate“ wäre ein Front-of-Neck Access die alternative Möglichkeit zur Sicherung des Atemwegs gewesen. Dies wurde antizipiert und in Tracheotomiebereitschaft intubiert.

## Besonderheiten des Falls

- Eine Verletzung durch einen Roboterarm ist ein seltener Mechanismus, das Verletzungsmuster war unvorhersehbar, der 2 kg schwere Roboterarm war in situ.
- Sitzende Position: Der Patient tolerierte aufgrund der Schmerzen und des penetrierenden Fremdkörpers keine Lageänderung. Während für die elektive wache FOI die Extension des Kopfes empfohlen wird [16], zeigen Fallberichte, dass die wache FOI auch in unkonventionellen Positionen erfolgreich durchgeführt werden kann, zum Beispiel in Bauchlage [17]. Dies demonstriert die Flexibilität und Anpassungsfähigkeit der wachen FOI an schwierige klinische Situationen.
- Unkooperativer Patient: Die psychische Anspannung und Unkooperativität des Patienten stellten eine zusätzliche Herausforderung dar. Obwohl fehlende Patientenkooperation für die wache FOI eine relative Kontraindikation darstellt [15], konnten durch adäquate Topikalisierung und Führung des Patienten eine erfolgreiche Intubation erreicht werden.

## Fazit für die Praxis

- Dieser Fall demonstriert die erfolgreiche Anwendung der wachen nasalen FOI bei einem Patienten mit schwerer penetrierender Gesichts‑/Halsverletzung.
- Die wache FOI ermöglicht eine Atemwegssicherung unter Erhaltung der Spontanatmung und der Schutzreflexe.
- Um diese Technik, die zu Recht als unverzichtbar für das sichere Management des erwartet schwierigen Atemweges bezeichnet wird, auch im Notfall beherrschen zu können, muss diese regelmässig praktiziert werden.

## Data Availability

Alle dieser Arbeit zugrunde liegenden Daten sind in diesem Artikel enthalten.
